# Convection-Enhanced Delivery of a First-in-Class Anti-β1 Integrin Antibody for the Treatment of High-Grade Glioma Utilizing Real-Time Imaging

**DOI:** 10.3390/pharmaceutics13010040

**Published:** 2020-12-30

**Authors:** Chibueze D. Nwagwu, Amanda V. Immidisetti, Gabriela Bukanowska, Michael A. Vogelbaum, Anne-Marie Carbonell

**Affiliations:** 1 Emory University School of Medicine, Atlanta, GA 30322, USA; cdnwagw@emory.edu; 2 Rutgers Robert Wood Johnson Medical School, New Brunswick, NJ 08901, USA; avi6@rwjms.rutgers.edu; 3 OncoSynergy, Inc., Stamford, CT 06902, USA; gabriela@oncosynergy.com; 4 H.Lee Moffitt Cancer Center and Research Institute, Departments of Neurosurgery and Neuro-Oncology, Tampa, FL 33612, USA; Michael.Vogelbaum@moffitt.org

**Keywords:** glioblastoma, high-grade glioma, convection enhanced delivery, OS2966, CD29, β1 integrin, ITGB1, monoclonal antibody, clinical trial

## Abstract

Introduction: OS2966 is a first-in-class, humanized and de-immunized monoclonal antibody which targets the adhesion receptor subunit, CD29/β1 integrin. CD29 expression is highly upregulated in glioblastoma and has been shown to drive tumor progression, invasion, and resistance to multiple modalities of therapy. Here, we present a novel Phase I clinical trial design addressing several factors plaguing effective treatment of high-grade gliomas (HGG). Study Design: This 2-part, ascending-dose, Phase I clinical trial will enroll patients with recurrent/progressive HGG requiring a clinically indicated resection. In Study Part 1, patients will undergo stereotactic tumor biopsy followed by placement of a purpose-built catheter which will be used for the intratumoral, convection-enhanced delivery (CED) of OS2966. Gadolinium contrast will be added to OS2966 before each infusion, enabling the real-time visualization of therapeutic distribution via MRI. Subsequently, patients will undergo their clinically indicated tumor resection followed by CED of OS2966 to the surrounding tumor-infiltrated brain. Matched pre- and post-infusion tumor specimens will be utilized for biomarker development and validation of target engagement by receptor occupancy. Dose escalation will be achieved using a unique concentration-based accelerated titration design. Discussion: The present study design leverages multiple innovations including: (1) the latest CED technology, (2) 2-part design including neoadjuvant intratumoral administration, (3) a first-in-class investigational therapeutic, and (4) concentration-based dosing. Trial registration: A U.S. Food and Drug Administration (FDA) Investigational New Drug application (IND) for the above protocol is now active.

## 1. Introduction

High-grade gliomas (HGGs) continue to be among the most formidable cancer diagnoses and highlight the unmet need for effective treatments. They include WHO Grade III gliomas (anaplastic astrocytoma, anaplastic oligodendroglioma, and malignant glioma) as well as Grade IV (glioblastoma). They are the most common and most malignant primary brain tumors and are associated with high morbidity and mortality [1]. Of these, glioblastoma is the most common and accounts for 48.3% of all gliomas as well as 14.6% of all primary brain tumors [1]. Despite standard of care therapy (which includes maximal surgical resection, radiotherapy, and chemotherapy), most of these tumors recur within 6 to 9 months and the median overall survival remains at 15 months [2].

There are several therapeutic challenges to be considered when treating HGG. First, the blood–brain barrier (BBB) can complicate the delivery of therapeutics to the site of the disease. The BBB excludes 100% of large molecule therapeutics and over 98% of small-molecule drugs, significantly limiting the range of pharmaceutical interventions that can be used in the central nervous system (CNS) [3]. Since most systemically administered therapeutics will not make it into the CNS, further efforts to bypass the BBB are warranted if these therapies are to be used for intracranial indications. In the event that the therapeutic agent in question is a small molecule that is able to penetrate the BBB, when administered systemically, the dose required to achieve therapeutic concentrations in the tumor microenvironment often causes systemic toxicity, limiting the safety, tolerability, and feasibility of these therapies. Furthermore, HGGs are highly infiltrative into the surrounding parenchymal brain tissue, limiting the therapeutic effect of surgical resection. Even if the contrast-enhancing tumor is maximally resected, tumor stem cells that invade the surrounding tissue persist, inevitably leading to a recurrence of the disease. Most of these tumors recur within 2 cm of the original site of disease [4]. Due to the heterogenous nature of these tumors, therapeutic resistance is encountered ubiquitously. This array of challenges highlights the need for novel therapeutic approaches in the treatment of HGG.

Here, we present an innovative framework for an active clinical trial using a novel therapeutic candidate, OS2966, in combination with a direct delivery method to address each of these therapeutic challenges. OS2966 is an anti-CD29/β1integrin/ITGB1 monoclonal antibody (mAb) that targets the entire family of β1 subunit- containing integrins. These receptors have been implicated in several hallmarks of cancer including growth, proliferation, invasion, angiogenesis, immune response, and therapeutic resistance [5]. By blocking this class of cell surface receptors, multiple mechanisms that drive malignancy can be attenuated simultaneously. The use of convection-enhanced delivery (CED) to deliver OS2966 directly to the site of disease bypasses the BBB entirely, ensuring that therapeutic concentrations are achieved in the CNS. CED produces a pressure gradient to deliver therapeutics at both greater rates and volumes than by diffusion alone [6]. Furthermore, co-infusion of OS2966 with a gadolinium chelate contrast agent will allow for real-time magnetic resonance imaging (MRI) visualization to ensure that the infusate reaches the targeted region of brain.

The purpose of this article is to provide an overview of the unique elements of this Phase I first-in-human clinical trial including: (1) the background and proposed mechanism of the novel therapeutic candidate OS2966, (2) a combinatory approach using CED for direct delivery, (3) the co-convection of gadolinium contrast enabling real-time MRI-visualization of infusion, (4) a two-part study design allowing for tissue sampling pre-and post-treatment, and (5) concentration-based dose escalation. Additionally, we discuss future considerations for this treatment paradigm in the setting of HGG.

## 2. Materials, Method, and Rationale

### 2.1. OS2966

OS2966 is a first-in-class, humanized and deimmunized anti-CD29/β1integrin/ITGB1 monoclonal antibody (mAb). Critical pathways that are governed by CD29 (β1 integrin subunit) signaling include the hallmarks of cancer, which are biological capabilities acquired during the multistep development of human tumors. These include growth factor signaling (survival/proliferation), invasion and metastasis (cell motility), angiogenesis and vascularization, immune response (inflammation, immune evasion), and resistance to conventional therapy (increased survival, epithelial-to-mesenchymal transition, enhanced stress responses) [5].

CD29 (β1 integrin) has been demonstrated to be upregulated in glioblastoma cells both in vitro and in vivo [7,8,9]. Further, Carbonell et al. (2013) demonstrated β1 integrin blockade produces antiproliferative, anti-invasive, antivascularization, proapoptotic, and antiresistance properties in glioblastoma cells resulting in dramatic growth suppression and/or complete regression in xenograft models [10].

OS2966 demonstrated exceptional therapeutic potential in preclinical studies and has been granted both investigational new drug status and orphan drug designation by the United States Food and Drug Administration (FDA) for the treatment of high-grade glioma in clinical trials.

**Figure 1:**
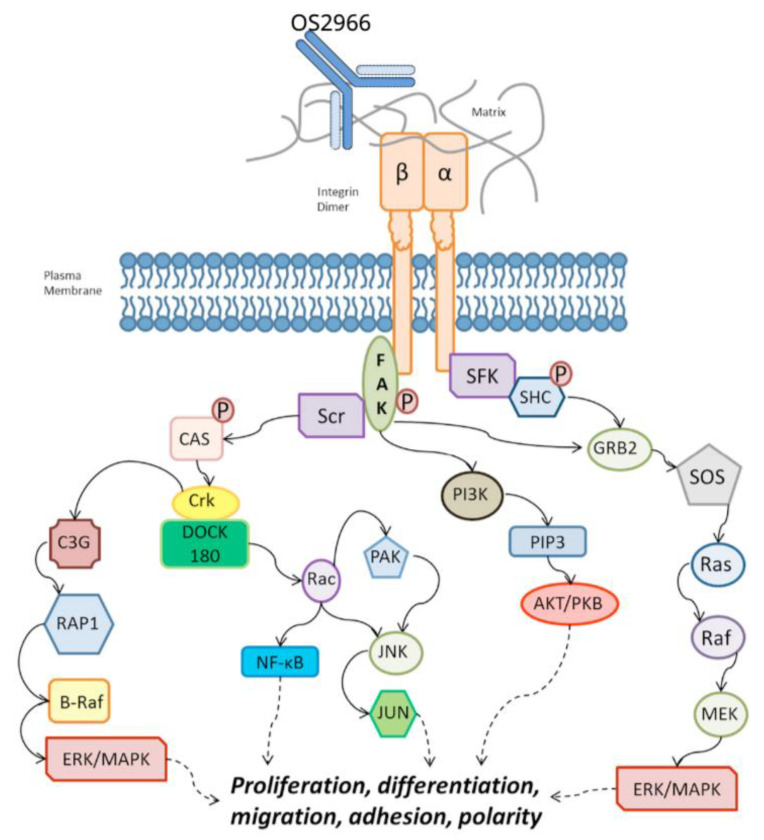
This illustrates the various pathways downstream of the β-1 integrin/CD29 receptor. Critical pathways that are governed by CD29 (β1 integrin subunit) signaling include the hallmarks of cancer, biological capabilities acquired during the multistep development of human tumors: growth factor signaling (survival/proliferation), invasion and metastasis (cell motility), angiogenesis and vascularization, immune response (inflammation, immune evasion), and resistance to conventional therapy (increased survival, epithelial-to-mesenchymal transition, enhanced stress responses). OS2966 is a first-in-class humanized and deimmunized anti-CD29 monoclonal antibody (mAb). Adapted with permission from [11], Frontiers Media S.A., 2014.

Figure 1 showing OS2966 mechanism of action.

### 2.2. Convection Enhanced Delivery and Infuseon Cleveland Multiport Catheter

Convection-enhanced delivery (CED) is a direct delivery method that can be used to bypass the BBB. Its advantages include delivery of high local concentrations of therapeutics with limited systemic exposure (thus, reduced risk of systemic toxicity). One or more CED catheters are sterotactically placed into a targeted area of brain (either the contrast-enhancing portion of a tumor or into the surrounding non-enhancing tumor-infiltrated brain parenchyma). Consequently, CED further exploits the extracellular interstitial space to achieve a constant, low-pressure infusion. Previously, “off-the-shelf” catheters had been repurposed for the CED of therapeutics in glioblastoma patients; however, these catheters were found to be prone to complications such as infusate backflow around their outer surface and blockage or plugging during insertion into the brain [11]. Infusate reflux is a critical issue because its presence substantially counteracts the goal of CED-homogeneous distribution of infusate through the extracellular space by bulk flow.

To combat the limitations associated with repurposed “off-the-shelf” catheters, specialized catheters such as the Infuseon Cleveland Multiport Catheter ™ (ICMC) were designed. Specifically, the ICMC was designed to meet the following performance criteria: (1) capability to perform reliable, high-volume delivery into brain tumors and brain parenchyma; (2) can be left in place for several days at a time; (3) constructed of materials that are biocompatible and compatible with a wide range of potential therapeutics, including biologics; (4) can be placed with the use of conventional stereotactic neurosurgical techniques; (5) compatible with use in an MRI environment; and (6) can be visualized with computerized tomography (CT) [12].

The ICMC’s key design elements combat ineffective infusate delivery by addressing lumen blockage and reflux. The ICMC consists of a central catheter shaft that houses 4 independent lumen microcatheters. This mitigates the risk of blockage or plugging of the catheter during placement or infusion, as the patency of each microcatheter lumen is unaffected by potential blockages in the others. Additionally, these catheters are retracted and protected within the central shaft during surgical placement and are not exposed until the infusion is about to begin. At that time, the stylet is removed and causes these 4 microcatheters to deploy radially, further ensuring maximal coverage of the targeted region of brain.

The ICMC has been in clinical use since December 2014 and has been employed in 3 clinical pilot studies to deliver therapeutics directly to tumor tissue or to the surrounding infiltrated brain parenchyma in patients with recurrent HGG (NCT02278510, NCT02500459, and NCT03193463) [12]. In these studies, the ICMC has been successfully used in the operating room (OR), within the MRI environment, and for several days outside of the operating room.

Another major advancement in the safe and potentially efficacious use of CED has been the development of real-time CED, which utilizes MRI to visualize the entire infusion process with the aid of co-convected tracers such as Gadoteridol (Prohance^®^). The use of real-time CED allows physicians to directly visualize and monitor the distribution of therapeutics within the brain. Therefore, potential infusate reflux along the CED catheter or leakage outside the target area can be detected and corrected in real-time (e.g., by altering the infusion rate). Furthermore, the ability to monitor distribution of infusate in real-time mitigates the historical challenge of potential variations in catheter placement between investigators and allows for adjustments in the infusion parameters if required. Specifically, with co-convection of contrast, we can confirm maximal tumor coverage is achieved regardless of technique variations between investigators. Vogelbaum et al., 2019, studied the delivery characteristics of the ICMC, which we expect to be reproducible in this study. These techniques will be used in the current study to ensure OS2966 is effectively delivered directly to the site of disease, thus conferring increased safety and optimized likelihood of clinical efficacy.

### 2.3. Study Population

This study is indicated for patients aged ≥ 18 years with histologically confirmed diagnosis of a stereotactically accessible, supratentorial, contrast-enhancing WHO Grade III or IV glioma (HGG) with a maximum volume between 2 and 6 cm^3^. Patients must have completed standard of care chemoradiation and have evidence of tumor recurrence or progression based on imaging studies within the previous 21 days that supports a clinically indicated resection. Additional inclusion criteria will include Karnofsky Performance Status (KPS) ≥ 70, adequate bone marrow, hepatic, renal, and coagulation functions; negative beta-human chorionic gonadotropin serum pregnancy tests, and adherence to oral contraceptive therapy in women of child-bearing age.

The exclusion criteria as it pertains to the patient’s HGG presentation will include the presence of any of the following: multicentric disease, tumor extending to opposite cerebral hemisphere; subependymal or leptomeningeal tumor dissemination; tumor located in the posterior fossa; or significant mass effect. Other exclusion criteria will include present history of other significant medical illness; history of HIV/AIDS; present history of active infection; history of hypersensitivity to gadolinium contrast agents; participation in another investigational drug trial in the past month; or patients currently receiving anticoagulants, antiplatelets, nonsteroidal anti-inflammatory drugs (NSAIDs), or escalating doses of steroids.

### 2.4. Study Site

Study participants will be assessed and treated at Moffitt Cancer Center in Tampa, FL, USA. There are plans in place to expand the trial to other study sites.

### 2.5. Study Design

This Phase I clinical trial is a single-center, ascending-dose, open-label, 2-part study designed to determine the safety and tolerability of OS2966, as well as the optimal infusion parameters when administering OS2966 intratumorally and intraparenchymally by CED in patients with recurrent/progressive HGG undergoing a clinically indicated surgical resection. OS2966 will be delivered by CED using the ICMC in both parts of the study.

Patients enrolled in this study will undergo 2 staged parts of treatment. In Study Part 1, patients will receive a single intratumoral infusion of OS2966 directly to the contrast-enhancing bulk tumor (“intratumoral administration”) by CED using the ICMC. During this portion of the study, the maximum duration of infusion will be 4 h as the infusion will take place while the patient is under anesthesia. In Study Part 2, the same patients will undergo a clinically indicated surgical resection of the previously infused tumor within 1 to 10 days (optimally within 1 to 3 days) following completion of the first OS2966 infusion. Immediately following surgical resection, 2 ICMCs will be placed directly into the surrounding non-enhancing tumor-infiltrated brain, and perioperative infusion of OS2966 will take place over a 4 h period (“intraparenchymal administration”). To confirm the overall quality of OS2966 delivery, a 2-mM concentration of gadoteridol (ProHance^®^, a gadolinium contrast agent used to visualize the infusion) will be added to OS2966 before each infusion in order to enable real-time image guidance during the infusion procedures. All patients will be closely monitored both clinically and through the use of imaging assessments. All enrolled patients will also receive standard supportive care therapy.

Safety will be assessed by evaluating adverse events (AEs), KPS, clinical laboratory examination results, vital sign measurements, 12-lead echocardiogram (ECG), and physical examination findings. Efficacy will be assessed by tumor response, defined by the Response Assessment in Neuro-Oncology (RANO) criteria; PFS; and durable response rate (DRR), defined as the percentage of patients with a complete response (CR) or partial response (PR) that is continuously maintained for ≥ 6 months. A blood sample will be obtained before and after each OS2966 infusion for pharmacokinetic assessment to determine whether OS2966 is present systemically after localized direct delivery to the brain.

This trial was approved by both the institutional review board and scientific review committee at Moffitt Cancer Center. The trial is registered with the national clinical trials database at ClinialTrials.gov (reference number NCT04608812).

**Figure 2:**
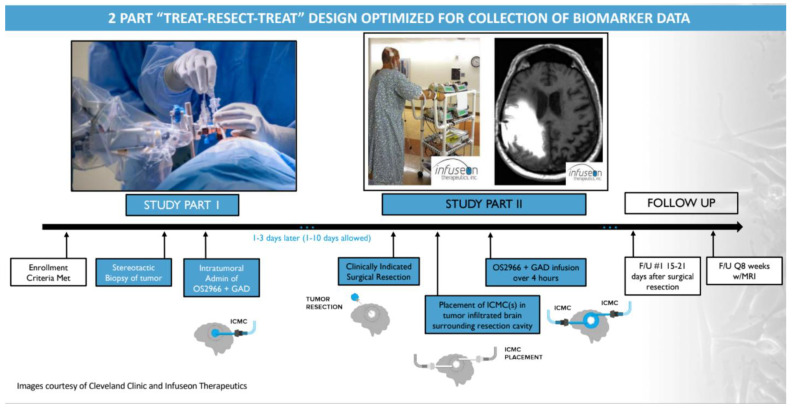
This schematic depicts the 2-staged approach with OS2966 treatment. In Study Part 1, patients will receive a single intratumoral infusion of OS2966 directly to the contrast-enhancing bulk tumor (“intratumoral administration”) by CED using the ICMC. In Study Part 2, the same patients will undergo a clinically indicated surgical resection of the previously infused tumor within 1 to 10 days (optimally within 1 to 3 days) following completion of the first OS2966 infusion. Immediately following surgical resection, 2 ICMCs will be placed directly into the surrounding non-enhancing tumor-infiltrated brain, and perioperative infusion of OS2966 will take place over 4 h (“intraparenchymal administration”). To confirm the overall quality of OS2966 delivery, a 2-mM concentration of gadoteridol (ProHance^®^, a gadolinium contrast agent used to visualize the infusion) will be added to OS2966 before each infusion in order to enable real-time MRI use during the infusion procedures. F/U = follow up; GAD = gadolinium; ICMC = Infuseon Cleveland Multiport Catheter.

An overall study timeline/schema is presented in Figure 2 below.

### 2.6. Rationale for Study Design

#### 2.6.1. Rationale for Two-Part Study Design

The study is divided into two parts, primarily to obtain pertinent pharmacokinetic (PK) and pharmacodynamic (PD) data that will be critical in determining the optimal biological dose of OS2966. In terms of pharmacokinetics, the 2-stage design allows for the determination of how OS2966 distributes in both the bulk contrast-enhancing tumor (intratumoral administration) and in the surrounding tumor-infiltrated brain (intraparenchymal administration) when delivered by the ICMC. The data obtained (e.g., volume of distribution/volume of infusion ratio) will allow for the determination of optimal CED infusion parameters when using the ICMC, including infusion rate and infusion duration in order to maximize the volume of distribution—a secondary study objective. This is key to the clinical development of OS2966 and will allow delivery of OS2966 with increased accuracy to both locations (bulk contrast-enhancing tumor and surrounding tumor-infiltrate brain) that have distinct differences in tissue characteristics. This design also allows for collection of post-infusion tissue from surgical resection in order to explore pharmacodynamic parameters. Specifically, the study aims to confirm that the antagonism of CD29 leads to downregulation and decreased CD29 expression when compared to matched pre-infusion samples. PD analysis also allows for the verification of the OS2966 mechanism of action by evaluating downstream signaling pathways.

#### 2.6.2. Rationale for Concentration-Based Dosing

Traditional evaluation of drug-induced toxicity has been primarily dose-oriented. This approach applies best for systemic intravascular delivery, where therapeutic agents are transported away from the site of injection via blood flow and distribute throughout the body in a roughly uniform manner. Ultimately, “dose” is a surrogate for tissue concentration, and the frequent use of body surface area (BSA) for dosing reflects the fact that the impact of a dose depends upon the volume of tissue to which it is distributed. Conversely, with local drug administration directly to brain tumors and brain parenchyma, the concept of “dose” does not accurately describe the actual tissue concentration, which is the most relevant factor that impacts efficacy and toxicity. Instead, the tissue concentration is a function of the drug concentration in the infusate and the degree to which the infusate disperses within the target tissue (V_d_).

When maximum coverage of brain tumors or tumor-infiltrated brain is the objective, concentration again becomes more important than “dose” for practicality reasons. Tumor volumes vary; therefore, defining a dose in the traditional form would limit coverage and consequently efficacy. The exception to this is in the case of therapeutics that do not necessarily require maximum coverage for efficacy (such as replicating oncolytic viruses or immunotherapies).

**Figure 3:**
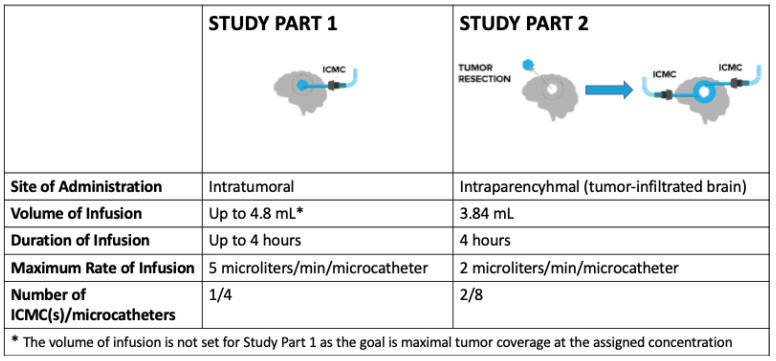
This dynamic two-part study design allows for variations in volume and rate of infusion while the concentration at the assigned dose level is held constant. This allows investigators to adjust infusion parameters for tumors of various sizes and physical characteristics such that they are uniformly infused with the same concentration of OS2966.

Because the tumor volume of each enrolled patient varies, and the objective is maximal tumor coverage, a fixed volume of infusion (and thus “dose” in traditional terms) is not practical and will not result in uniform tissue concentration. Instead, a fixed concentration of OS2966 infusate and a concentration-based dose escalation design allows for consistent tissue concentration among varying tumor volumes, thereby increasing patient eligibility by allowing the study to accommodate patients with a broader range of tumor sizes. This dynamic study design allows for variable volume and rate of infusion while the concentration delivered remains constant (Figure 3). Consideration of patient eligibility is important as, according to a keynote presentation given by Dr. John Sampson on 17 November 2018 at the annual Society of Neuro-Oncology (SNO) meeting, approximately only 10% of patients in the recurrent glioblastoma population are participating in clinical studies despite the grave prognosis.

### 2.7. Objectives & Outcome Measures

The primary objectives of this study include assessment of safety and tolerability of OS2966, and determination of the optimal biological dose of OS2966. These will both be evaluated when OS2966 is administered intratumorally and intraparenchymally.

The secondary objectives involve determination of optimal CED infusion parameters using the ICMC, determination of systemic exposure (if any) to OS2966, and assessment of the preliminary efficacy of OS2966 when delivered directly to the tumor-infiltrated brain by CED using the ICMC.

Additional exploratory objectives will include the further characterization of the PK and PD effects of OS2966, and the assessment of additional safety parameters such as vital signs, 12-lead ECG, physical examination, KPS scores, clinical laboratory assessments, and AEs by system organ class, severity, and seriousness.

Study objectives will be evaluated throughout the study as defined in Table 1.

### 2.8. Dose Escalation: Accelerated Titration Design

This study uses CED as a delivery method; therefore, the local tissue concentration of OS2966 is a more relevant metric than the traditional “dose” reported in total milligrams delivered. The use of concentration-based dosing accounts for tumor variation between patients. For example, an equal “dose” in milligrams administered to two tumors of different volumes would not achieve the same tissue concentration. Thus, patients will be sorted into dose levels defined by concentration to be delivered. Since patients may receive varying volumes at the pre-defined concentration to achieve maximal tumor coverage as determined by the investigator, the “absolute dose” will be a calculated value that depends on the product of the concentration and total volume delivered.

**Figure 4:**
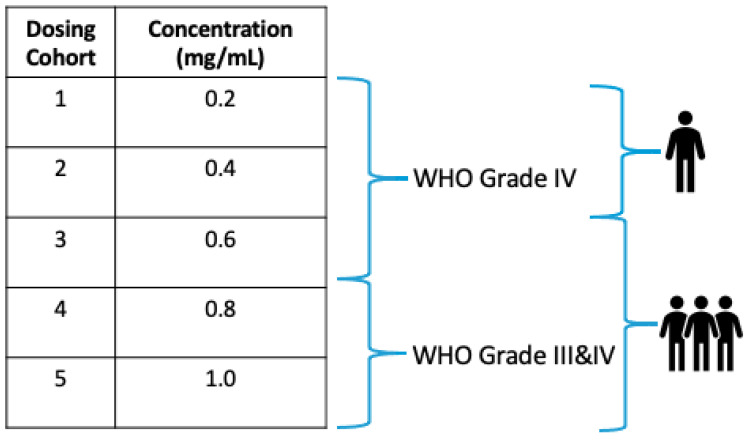
Dose escalation will be conducted at five pre-determined concentration levels. The first two dosing cohorts will enroll one patient each. Starting at the third dose level, the study will convert into a “3 + 3” design, in which three patients will be enrolled into each dosing cohort. If any qualifying adverse events (AEs) or dose-limiting toxicity (DLTs) occur in the first two cohorts, the study will automatically convert to “3 + 3” at that dose level.

Five concentration levels (dosing cohorts) were defined using a modified Fibonacci sequence and are further described in Figure 4. The same dosing cohorts will be used for both Study Parts 1 and 2. Specifically, each patient will receive the same assigned concentration of OS2966 in both parts of the study.

**Figure 5:**
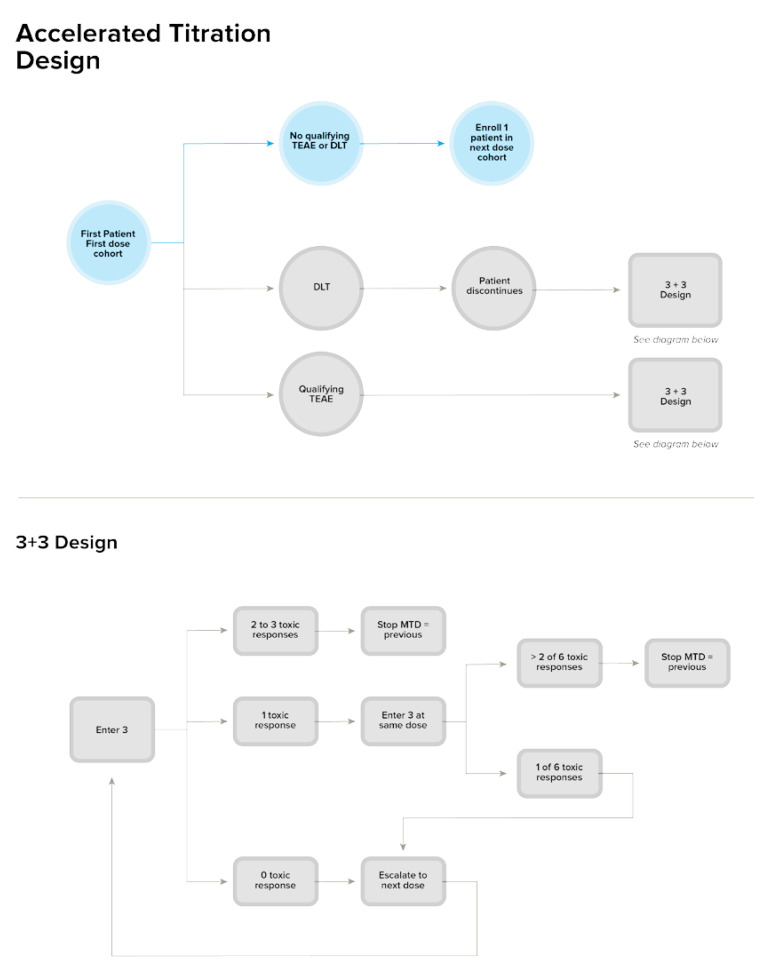
Detailed Flow Diagram of the Accelerated Titration Design. a = Applicable to dose concentration level 1 and 2 only. b = Dose escalation for dose concentration levels 3, 4, 5 will follow the depicted 3 + 3 design only. DLT = dose-limiting toxicity; MTD = maximum tolerated dose; TEAE = treatment-emergent adverse event.

Given the nonclinical safety profile of OS2966, and the fact that recurrent/progressive HGG is a serious condition with high unmet medical need, dose escalation will follow a rule-based accelerated titration design (ATD) for the first 2 dose concentration levels. The ATD allows fewer patients to be treated at potentially subtherapeutic doses, reduces study duration, and ensures collection of important information that is needed to plan for phase 2 studies. To capture data from an adequate number of patients at higher (and potentially efficacious) dose levels, dose escalation will convert to a standard 3 + 3 design starting with dose cohort 3. The planned ATD is further illustrated in Figure 5.

For the initial 2 dose concentration levels, single-patient cohorts will be enrolled. If a patient in the first 2 dose concentration level experiences a qualifying AE that is possibly related to study treatment (OS2966 infusion by the ICMC), or 1 dose-limiting toxicity (DLT) occurs, the study will adapt to a standard 3 + 3 dose-escalation design at the same defined concentration levels. As mentioned above, starting at the third dose concentration level, the study will automatically convert to a standard 3 + 3 design [13].

### 2.9. Safety Evaluation Windows

As this study involves surgical procedures in close proximity to OS2966 infusion(s), 3 windows, or safety evaluation periods have been defined to ensure appropriate attribution to any observed toxicity (e.g., to OS2966 infusion, surgical resection, etc.). These safety evaluation periods are consistent with those described by Kunwar et al., 2006, where 3 symptomatic windows were defined: the first window occurs between the surgical procedure and CED, the second occurs during CED and up to 1 week after its completion, and the third window occurs 2 to 10 weeks after treatment [14]. These windows were reported to generally reflect AEs related to surgical procedures, mass effect from infusate, and drug effect on tumor-infiltrated and normal brain parenchyma, respectively.

#### 2.9.1. Safety Evaluation Period 1: First OS2966 Infusion (Intratumoral) up to Clinically indicated Surgical Resection

During this evaluation period, patients will undergo a stereotactic biopsy, followed by ICMC placement and intratumoral infusion of OS2966 under real-time imaging observations of gadoteridol distribution in order to ensure accuracy of OS2966 delivery. Therefore, neurologic AEs during this safety evaluation period could be attributed to the stereotactic biopsy, ICMC placement, or the OS2966 infusion. These AEs can be separated both temporally and based on imaging.

Adverse neurologic events related to stereotactic biopsy or ICMC placement (e.g., intracranial hemorrhage, focal neurologic deficits, etc.) would present immediately (within 24 h) post-procedure and would be localized to the site of the catheter placement or biopsy on imaging. It is important to note that clinical experience with the ICMC (and other CED catheters) demonstrates that the procedure surrounding catheter placement is extremely safe. In fact, no surgical complications related to catheter placement using the ICMC, which limited infusion of the investigational product, have been reported in either the currently available published clinical literature [11], or in any of the ongoing clinical studies with the ICMC (NCT02500459, NCT03193463).

Adverse neurological events related to intratumoral OS2966 infusion would most commonly be caused by potential mass effect from the infusate. Mass effect peaks at 48 h (postinjury or post-infusion in this case) and is often responsive to corticosteroids [14]. Mass effect typically presents with signs of increased intracranial pressure (e.g., headache, nausea, vomiting, lethargy) or focal neurological deficits and can be confirmed on T2-weighted MRI as abnormal hyperintensity. It is important to note that any impact OS2966 has on the tumor itself (e.g., significant mass effect, delayed intratumoral hemorrhage, etc.) will be mitigated by subsequent resection of the tumor (Study Part 2, Safety Evaluation Period 2), optimally done within the same hospitalization.

#### 2.9.2. Safety Evaluation Period 2: Clinically Indicated Surgical Resection to Full Postoperative Recovery

During this evaluation period, patients will undergo surgical resection of their previously infused tumor followed by ICMC(s) placement in the surrounding tumor-infiltrated brain. No OS2966 will be administered during this period, and the first dose of OS2966 will essentially be removed during tumor resection. Therefore, neurologic AEs during this safety evaluation period would be attributed to the standard of care surgical resection or ICMC placement, and if significant, would be detected on neurological examination and or imaging at the time of full postoperative recovery (prior to the second OS2966 infusion).

#### 2.9.3. Safety Evaluation Period 3: Second OS2966 Infusion (Perioperative Intraparenchymal) through 28-Day Dose-limiting Toxicity Observation Period

During this evaluation period, patients will undergo perioperative intraparenchymal infusion of OS2966 to the surrounding tumor-infiltrated brain over a four-hour time period. OS2966 infusion-related neurological AEs occurring during this safety evaluation period would most commonly reflect mass effect from the infusate (time of onset typically peaking again at 48 h and seen up to 7 days after completion of infusion) [13]. Furthermore, the patient will be awake during this safety evaluation period and full neurological examination can be performed; therefore, if signs of mass effect occur (e.g., focal neurological deficits, signs of elevated intracranial pressure) the infusion may be stopped and or the rate of infusion reduced. If this ameliorates the symptoms, the AE will be clearly attributable to the OS2966 infusion. After the infusion is complete and discharge criteria are met, patients will follow up 15 to 21 days after their surgical resection for suture removal and a safety assessment. At this time, a neurologic examination will be performed, and MRI will be obtained to assess for any delayed AEs related to OS2966. Given the nonclinical toxicity profile of OS2966 we do not anticipate any delayed toxicity; however, patients will be closely observed for signs of toxicity through the dose-limiting toxicity (DLT) period and beyond.

## 3. Stopping Rules

Stopping rules for halting the study, individual treatment, and/or dose escalation have been specified in the study protocol. Criteria were established to protect patient safety and take any potential treatment-related toxicities into consideration. In the event that the study meets criteria for these stopping rules, it will only be restarted upon investigator and/or SRC approval.

## 4. Summary of Potential Risks and Benefits

Given this is a Phase 1 trial, patients may not derive any benefit from study treatment; however, it is also possible that patients with recurrent/progressive HGG may experience stable disease or partial or complete response, which may lead to longer PFS, as a result of treatment with OS2966.

The potential risks of study participation include those associated with exposure to OS2966 and the risks of surgical biopsy, tumor resection, and study drug administration using CED via the ICMC.

OS2966 has been studied in four nonclinical, nonhuman primate (NHP) studies. No investigational treatment-related AEs were observed in the intracerebral (IC) study where OS2966 was delivered by CED to the normal CNS (right corona radiata) of NHP under real-time MRI. Results of the histopathological analysis of NHP brain tissue following IC administration of OS2966 revealed the presence of OS2966-related perivascular mononuclear cell infiltrates and perivascular cuffing, which is an indication of inflammation within the target structure. Immunohistological staining against markers for activated glial and microglial cells confirmed the activation of localized neuroinflammation within the injected side of the brain. The marker activation was dose and time dependent, indicating that the inflammation was likely transient in nature and driven by catheter placement and the presence of a humanized protein that is foreign to NHPs. This response may be attenuated in humans, as the mAb was designed to display increased similarity to antibodies produced by humans and is therefore expected to be less immunogenic in this setting. No systemic exposure of OS2966 was observed following intracerebral administration. OS2966 was not detected in the serum at any time point by enzyme-linked immunosorbent assay [ELISA]).

As noted above, the findings of toxicity studies in NHPs revealed that direct delivery of OS2966 to the brain by CED did not lead to systemic exposure. The aforementioned NHP studies were performed in animals with intact BBBs. As our study will be conducted in patients with HGG, the integrity of the BBB may be disrupted. With this in mind, further studies were conducted to confirm the safety profile of OS2966 in the event of extravasation into systemic circulation. Pharmacokinetic analysis will also be performed during the trial to confirm whether OS2966 does indeed reach the systemic circulation. A Good Laboratory Practice (GLP) repeated-dose systemic toxicity study was completed to evaluate the safety of systemically administered OS2966 (at 5, 15, and 30 mg/kg) compared to the vehicle controls. Following intravenous dosing, which exceeded the intracerebral doses by at least 100-fold, no mortalities, clinical signs, weight losses, ocular changes, effects on measured 12-lead ECG parameters, changes in respiratory rates, changes in coagulation or urinalysis parameters, or gross findings at necropsy were observed. At doses higher than 5 mg/kg, marginal to slight decreases in total protein and albumin were noted. Additionally, slight changes in red-cell mass, reticulocytes, and platelets were noted as minor and reversible at doses higher than 5 mg/kg.

The results from the high-dose group (30 mg/kg) included a decreased red blood cell (RBC) mass associated with a regenerative increase in reticulocytes, decreased platelets, total protein, and albumin levels, as well as increased white blood cell counts (mainly lymphocytes and basophils). Patients will be monitored for such changes during clinical exposure to OS2966.

Risks related to the use of the ICMC catheter include displacement of the secured catheter, leakage of infusate from the catheter, and failure of the catheter to deliver therapy as intended.

Many of the procedures used in this study are standard of care and others are investigational. For standard of care procedures (e.g., craniotomy for tumor resection), there are risks independent of the investigational study.

## 5. Future Directions

### 5.1. Repeat Treatment with OS2966

This combinatory approach of OS2966 delivery via CED holds promise in the treatment of HGG. Should the desired outcome, clinical response to treatment, be achieved, there are several considerations to be made moving forward. If a cohort of treated patients does show either a partial or complete response, the optimal time to administer a repeat treatment cycle remains unclear. Options include: (1) preemptively re-dose responders prior to observation of radiographic progression, or (2) wait until radiographic progression is observed on follow-up imaging and re-treat at that time. In preclinical toxicology studies, NHPs were infused with OS2966 via CED. The presence of OS2966 was analyzed via immunohistochemical staining of the infused structures and were found to be positive on day six, but not on day 17, suggesting the clearance of OS2966 from parenchymal brain tissue at a timepoint within this range. Taken into context with PK data collected from this trial, this could guide the timing and approach to re-treat those who respond.

It is worth investigating the therapeutic effect of prolonging the first OS2966 infusion in future trials. Notably, Weber et al. (2003) and Bruce et al. (2011) were able to conduct CED over a period of 96 and 100 h, respectively [15,16]. Administering a longer course of OS2966 infusion initially may increase the duration of response and prolong the time until a second dose is needed.

Furthermore, the approach on whether or not to administer another treatment cycle to patients who do not exhibit a clinical response also remains unclear. The potential lack of response could be due to enrollment at a sub-therapeutic dose level, factors related to the specific patient’s tumor that require a higher dose or multiple cycles of treatment to exhibit response, or unknown genetic factors affecting tumor response.

Once the decision to administer another cycle of treatment has been made, the concentration of OS2966 to be administered must be determined. This can be guided by the frequency and severity of AEs experienced during the trial. This highlights the utility of using three separate safety monitoring windows, as it allows the study investigator to more accurately attribute AEs to the surgical resection, catheter placement, or to the infusion. Without this distinction, AEs due to standard of care tumor resection or catheter placement may be attributed to the infusion. In such a case, the rate of infusion or concentration of OS2966 may mistakenly be restricted, which unnecessarily limits treatment to a potentially sub-therapeutic dose.

### 5.2. Method of Delivery for Repeat Treatments

If clinical response is elicited with OS2966 treatment and additional cycles are to be given, further investigation into intracranial delivery of antibody therapies may be warranted. Until an implantable device that can be utilized to deliver chronic CED infusions is developed, subsequent treatments will remain a challenge. Such a device would be ideal, as this would circumvent the need for multiple procedures if further cycles of treatment are to be administered.

Of note, Vogelbaum et al. (2018) found that CED via the ICMC was safe and well tolerated in that study population and there were no adverse events attributed to catheter placement or infusion. Thus, it is not unreasonable to re-administer a subsequent round of OS2966 treatment via the ICMC in a similar fashion to what is planned in this Phase I study.

### 5.3. Long-Term Antibody Therapy

As with any mAb therapy, stable-long term expression of the protein in vivo would bypass the need for repeat dosing. This would significantly reduce both the risks and cost associated with repeated cycles of mAb treatment. In the case of HGG, stable long-term expression of the OS2966 antibody would eliminate the need for a surgical component to the treatment paradigm aside from initial tumor resection. As Samaranayake et al., 2009, pointed out, this may be achieved through administration of a gene carrying vector or through gene transfer [17]. Further preclinical studies are warranted to elucidate the feasibility of this approach to bring it from the lab bench to the bedside.

## Figures and Tables

**Table 1:** Objectives and outcome measures.

Evaluation of Objectives		
Objective	Endpoint	Analysis
**Primary**		
To determine the safety and tolerability of OS2966 when delivered both intratumorally (to the bulk, contrast-enhancing tumor) and intraparenchymally	Number of “qualifying” TEAEs or DLTs experienced by each patient.	The proportion of patients with at least 1 “qualifying” TEAE or DLT event will be reported using descriptive statistics.
To determine the OBD of OS2966 when delivered intratumorally/intraparenchymally	OBD	This will be determined based on the dosing protocols (ATD and 3 + 3)
**Secondary**		
To determine the optimal CED infusion parameters	1. Vd of OS2966 when delivered by the ICMC intratumorally (Study Part 1) and intraparenchymally (Study Part 2). 2. Relationship between Vi, infusion rate, and tumor coverage 3. Vd/Vi ratio.	ANOVA, ANCOVA, or polynomial regression analysis will be used to characterize these
To determine if OS2966 reaches the systemic circulation after direct delivery to the brain.	Concentration of OS2966 in blood pre and postinfusion of OS2966 in both Study Parts 1 and 2.	Descriptive statistics will be reported for PK blood samples collected pre and postinfusion of OS2966, as well as the change between the time points. The proportion of patients with detectable levels of OS2966 in their systemic circulation will be reported using descriptive statistics. A listing of concentration and other relevant covariates will be reported by patient.
To assess preliminary efficacy of OS2966 when delivered directly to the tumor-infiltrated brain by CED using the ICMC.	1. Tumor response, as measured on the RANO criteria. 2. Time to tumor progression, if noted during the study. An indicator variable (YES [Y]/NO [N]) for whether the patient’s response lasted at least 6 months. Those patients who do not respond at all (on the RANO scale) will be recorded as “N.”	1. Distribution of tumor responses among RANO categories by dosage group, and overall will be reported and described using descriptive statistics. A Fisher’s Exact Test will be used to assess the possibility of differences between the dosage groups, although a statistically significant result is unlikely in such a small study. 2. Time to tumor progression will be analyzed overall using a Kaplan-Meier analysis. Estimates for median, 25% quartile, 75% quartile, and survival plot will be provided for PFS. The DRR will be calculated and described using descriptive statistics.
**Exploratory**		
To further characterize the PK and to characterize the PD effects of OS2966 after intratumoral administration.	PK/PD assessment of OS2966	Pre and postinfusion CD29 RO will be summarized using descriptive statistics. Pre and postinfusion CD29 expression, as well as the change in expression, will be summarized using descriptive statistics.
To assess additional safety and tolerability parameters.	AEs by SOC	Safety and tolerability will be assessed by clinical review of all endpoints. Each endpoint will be summarized using descriptive statistics. The incidence, onset time, and titer of ADA will be summarized using descriptive statistics.

ADA = antidrug antibody; AE = adverse event; ANCOVA = analysis of covariance; ANOVA = analysis of variance; BP = blood pressure; CED = convection-enhanced delivery; DLT = dose-limiting toxicity; DRR = durable response rate; HGG = high-grade glioma; HR = heart rate; ICMC = Infuseon Cleveland Multiport Catheter; OBD = optimal biological dose; PD = pharmacodynamic; PFS = progression-free survival; PK = pharmacokinetics; RANO = Response Assessment in Neuro-Oncology criteria; RO = receptor occupancy; RR = respiratory rate; SOC = system organ class; TEAE = treatment-emergent adverse event; Vd = volume of distribution; Vi = volume of infusion.

## Data Availability

No new data were created or analyzed in this study. Data sharing is not applicable to this article.
